# Glibenclamide and HMR1098 normalize Cantú syndrome‐associated gain‐of‐function currents

**DOI:** 10.1111/jcmm.14329

**Published:** 2019-05-22

**Authors:** Marien J. C. Houtman, Xingyu Chen, Muge Qile, Karen Duran, Gijs van Haaften, Anna Stary‐Weinzinger, Marcel A. G. van der Heyden

**Affiliations:** ^1^ Division of Heart and Lungs, Department of Medical Physiology University Medical Center Utrecht Utrecht The Netherlands; ^2^ Department of Pharmacology and Toxicology University of Vienna Vienna Austria; ^3^ Center for Molecular Medicine, Department of Medical Genetics University Medical Center Utrecht Utrecht The Netherlands

**Keywords:** ABCC9, Cantú syndrome, electrophysiology, glibenclamide, HMR1098, pharmacology

## Abstract

Cantú syndrome (CS) is caused by dominant gain‐of‐function mutation in ATP‐dependent potassium channels. Cellular ATP concentrations regulate potassium current thereby coupling energy status with membrane excitability. No specific pharmacotherapeutic options are available to treat CS but I_KATP_ channels are pharmaceutical targets in type II diabetes or cardiac arrhythmia treatment. We have been suggested that I_KATP_ inhibitors, glibenclamide and HMR1098, normalize CS channels. I_KATP_ in response to Mg‐ATP, glibenclamide and HMR1098 were measured by inside‐out patch‐clamp electrophysiology. Results were interpreted in view of cryo‐EM I_KATP_ channel structures. Mg‐ATP IC_50_ values of outward current were increased for D207E (0.71 ± 0.14 mmol/L), S1020P (1.83 ± 0.10), S1054Y (0.95 ± 0.06) and R1154Q (0.75 ± 0.13) channels compared to H60Y (0.14 ± 0.01) and wild‐type (0.15 ± 0.01). HMR1098 dose‐dependently inhibited S1020P and S1054Y channels in the presence of 0.15 mmol/L Mg‐ATP, reaching, at 30 μmol/L, current levels displayed by wild‐type and H60Y channels in the presence of 0.15 mmol/L Mg‐ATP. Glibenclamide (10 μmol/L) induced similar normalization. S1054Y sensitivity to glibenclamide increases strongly at 0.5 mmol/L Mg‐ATP compared to 0.15 mmol/L, in contrast to D207E and S1020P channels. Experimental findings agree with structural considerations. We conclude that CS channel activity can be normalized by existing drugs; however, complete normalization can be achieved at supraclinical concentrations only.

## 1 INTRODUCTION

Cantú syndrome (CS) is a rare genetic autosomal dominant disorder, also known as hypertrichotic osteochondrodysplasia (MIM 239850), characterized by congenital hypertrichosis, distinctive facial features and cardiovascular defects.^1^, ^2^ CS patients are chronically ill; they suffer from severe phenotypes and possibly have a decreased life expectancy. CS is caused by dominant gain‐of‐function mutations in the ATP‐dependent potassium channel subunits *ABCC9* 4, 5 and *KCNJ8* 6, 7 encoding SUR2 and K_IR_6.1 respectively, responsible for I_KATP_ in many cell types. No specific (pharmaco)therapeutic options are currently available to treat CS.^8^

I_KATP_ channels are octamers consisting of four pore‐forming K_IR_6.x and four sulphonylurea receptors (SURs).^9^ *ABCC9* encodes SUR2A/B, mainly expressed in myocytes, skeletal muscles (SUR2A) and vascular smooth muscle cells (SUR2B),^10^ whereas the closely related *ABCC8* encodes SUR1 which is strongly expressed in pancreatic β‐cells. Intracellular ATP concentrations regulate the opening and closure of the I_KATP_ channels, thereby coupling cellular energy status with membrane excitability. By yet not fully understood mechanisms, ATP inhibits the channel directly by affecting the K_IR_6.x subunits, whereas Mg‐ATP and Mg‐ADP levels activate the channel by interaction with the nucleotide binding domains in the SUR subunits.^9^ CS‐associated mutations in SUR2 increase channel activity across a range of physiological intracellular nucleotide concentrations,^4^ thus making the channels less responsive to changes in intracellular energy levels, thereby decreasing cell excitability.

The I_KATP_ channel is a known pharmaceutical target for which both channel blocking and opening drugs are currently in development and used in the clinic.^11^, ^12^ For example, glibenclamide administration is widely used to treat type II diabetes, and has been highly effective in treating patients with neonatal diabetes caused by mutations in *ABCC8*. Glibenclamide inhibits the I_KATP_ channels in the pancreatic β‐cells, resulting in membrane depolarization and subsequent calcium‐channel activation. Increased intracellular calcium then induces insulin secretion from intracellular storage vesicles. Glibenclamide is rather unspecific and also strongly inhibits I_KATP_ channels consisting of SUR2 subunits present in myocytes.^8^ In contrast, HMR1098, the sodium salt of HMR1883, was shown to display enhanced specificity for SUR2‐ over SUR1‐based channels compared to glibenclamide,^14^ although SUR selectivity is disputed by more recent evidence.^15^ HMR1883/1098 has been developed to treat cardiac arrhythmias resulting from ischaemia‐induced cardiac I_KATP_ activation.^16^ In vivo, HMR1883 is able to counteract myocardial ischaemia‐associated ventricular fibrillation in conscious dogs and anaesthetized pigs by a mechanisms that probably includes normalization of refractory period, impulse propagation and ventricular activation.^17^, ^18^ Experimental studies revealed an interplay between sulphonylurea and Mg‐ATP mechanisms resulting in increased inhibition by the sulphonylurea at higher Mg‐ATP concentrations in K_IR_6.2/SUR1 channels, but not in K_IR_6.2/SUR2 channels.^20^, ^21^ Here, we have been suggested that HMR1098 and glibenclamide induce normalization of I_KATP_ channel activity in ectopic expression systems despite the presence of the CS‐associated *ABCC9* gain‐of‐function mutations.

## 2 MATERIALS AND METHODS

### 2.1 Constructs and mutagenesis

Nucleotide changes encoding the H60Y, D207E, S1020P and S1054Y were introduced into the pcDNA‐SUR2A (rat) expression construct 4 using the QuikChange II XL Site‐Directed Mutagenesis Kit (Stratagene) using custom‐designed mutagenesis primers (H60Y: gaagccatgtgttgtaatgaatttgcacttttgagctttggc; D207E: agcctccagaggaactccaggacctgg; S1016P (homologous to human S1020P): gtttatactgtactcgggggtccacgtagctagcc; S1050Y (homologous to human S1054Y): cattctacagtgaggtaggtgacgaggcaaagg). Sanger sequencing was performed to confirm the presence of introduced mutation. Generation of the R1154Q construct has been described earlier.^4^

### 2.2 Inside‐out I_KATP_ recordings

K_IR_6.2 subunits were used since these are the most amenable for studying SUR2 mutations in excised patch experiments and this also allows for comparison with other published in vitro studies of CS mutations. HEK293T cells were grown in DMEM medium supplemented with 2 mmol/L L‐Glutamine, Pen‐Strep (both 50 U/mL, Lonza, Breda, The Netherlands) and 10% FBS (Sigma‐Aldrich, Zwijndrecht, The Netherlands) at 37°C with 5% CO_2_. Three to four days prior to measurements, cells were put on Poly‐L‐Lysin (Sigma‐Aldrich)‐coated glass coverslips in a 24‐well plate and allowed to adhere for 24 hours. Subsequently transfection was performed with linear PEI with a molecular weight of 25 000 (Polysciences, Hirschberg an der Bergstrasse, Germany). PEI was prepared as described before.^22^ Briefly, 3.2 µg of pCMV6‐KCNJ11 (wild‐type), 3.2 µg of pCMV6‐SUR2A (wild‐type or mutant) and 1.6 µg of pEGFP‐C1 were added to 50 µL 1.5 mol/L NaCl and adjusted to 500 µL with H_2_O. Another 50 µL 1.5 mol/L NaCl was added to 64 µL PEI stock solution and adjusted to 500 µL with H_2_O. The PEI mixture was added to the DNA mixture and incubated for 20 minutes at room temperature. After incubation, 50 µL was added per well. Transfection was allowed to take place for at least 24 hours.

Patch‐clamp recordings were done using the excised inside‐out configuration. In order to obtain an inside‐out patch, a glass pipette (Harvard Apparatus, Holliston, USA) with a typical resistance of 1.5‐3.0 MΩ, filled with pipette solution (145 mmol/L KCl, 1 mmol/L CaCl_2_, 1 mmol/L MgCl_2_, 5 mmol/L HEPES, pH7.4 KOH), was placed on the cell and negative pressure was applied to achieve gigaseal. After the formation of gigaseal, the pipette was lifted and briefly exposed to air to allow inside‐out patch formation. The pipette was returned into the bath solution (131 mmol/L KCl, 1 mmol/L EGTA, 1 mmol/L MgCl_2_, 7.2 mmol/L K_2_HPO_4_, 2.8 mmol/L KH_2_PO_4_, pH7.2 KOH) and recording was started using an Axopatch 200B amplifier (Molecular Devices, San Jose, USA) and data were sampled at 20 kHz and filtered at 2 kHz. Using pCLAMP9 software (Molecular Devices), currents were recorded in voltage‐clamp mode using a repetitive pulse protocol (10 ms 0 mV, 1000 ms −40 mV, 25 ms −100 mV, 5 seconds ramp from −100 to + 100 mV, 3965 ms 0 mV). Inward and outward current levels were determined at −80 mV and +50 respectively after steady‐state was reached, and all recordings were performed at room temperature (20ºC) in a perfusion chamber.

In experiments where the IC_50_ value for Mg‐ATP was determined, the bath solution was supplemented with Mg‐ATP (Sigma‐Aldrich) at the following concentrations; 0.03, 0.1, 0.3, 1, 3 and 10 mmol/L yielding free Mg^2+^ concentrations of 0.96, 0.97, 0.99, 1.06, 1.21 and 1.61 mmol/L respectively as described before.^4^ Glibenclamide (Sigma‐Aldrich) and HMR1098 (Axon Medchem, Groningen, The Netherlands) stock solutions were made in DMSO and H_2_O respectively and diluted in bath solution to the desired concentrations. The IC_50_ for Glibenclamide was determined at 0.15 and 0.5 mmol/L Mg‐ATP and for HMR1098 at 0.15 mmol/L Mg‐ATP.

Rundown control experiments were performed and showed 8% ± 3% and 9% ± 3% (*P* < 0.05, n = 12) of inward and outward I_KATP_ current respectively after 10 minutes (Figure S1), which was also the time‐span of a typical experiment which included the full range of drug applications. IC_50_ values and Hill coefficients were calculated using GraphPad Prism 7 software and statistics were performed with Kaleidagraph software (Synergy Software, Reading, USA). All data are shown as ± SEM.

### 2.3 Cryo‐EM structures

Cryo‐EM structures of the I_KATP_ channels in different conformations (pdb: 6BAA, 3.63 Å resolution 23 and pdb: 6C3O, 3.9 Å 24) were energy minimized in two steps, using 5000 steps of steepest descent energy minimization, followed by 5000 steps of conjugate gradient energy minimization with the amber99sb forcefield in gromacs.^25^

## 3 RESULTS

### 3.1 Functional inhibition of CS‐associated I_KATP_ channel mutations by glibenclamide and HMR1098

**Figure 1:**
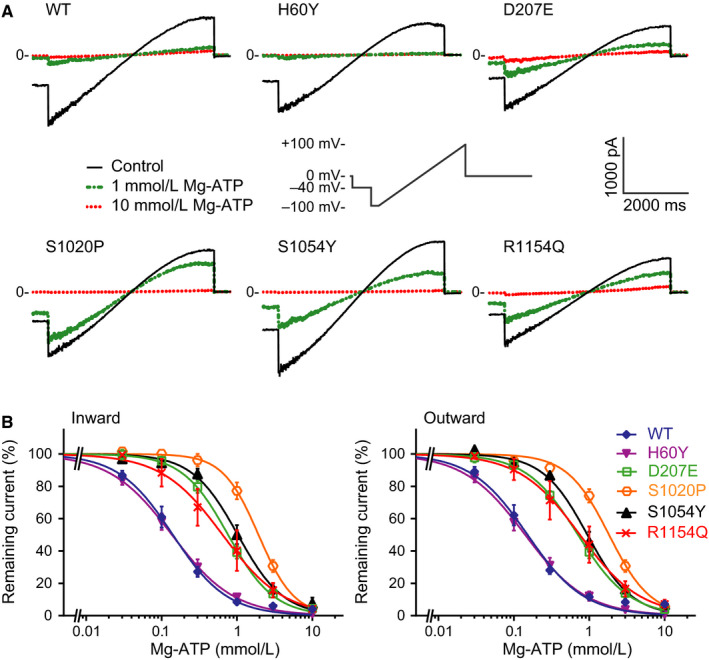
Dose‐dependent inhibition of K_IR_6.2/SUR2A wild‐type and mutant channels in response to Mg‐ATP. (A) Steady state K_IR_6.2/SUR2A current traces from SUR2A WT, H60Y, D207E, S1020P, S1054Y and R1154Q channel containing inside‐out patches elicited by a voltage ramp protocol from −100 to + 100 mV, under baseline conditions (c) and upon application of 1 or 10 mmol/L Mg‐ATP. Measurements were performed with symmetrical high‐potassium concentrations at both sides of the patch. Please note that the traces shown in panel A are an excised part, including 50 ms −40 mV, 5 s ramp from −100 to + 100 mV, 50 ms 0 mV, of the full trace. (B) IC_50_ curves of Mg‐ATP for the inward (at −80 mV) and outward (at + 50 mV) K_IR_6.2/SUR2A currents of SUR2A WT (n = 8), H60Y (n = 13), D207E (n = 7), S1020P (n = 11), S1054Y (n = 8) and R1154Q (n = 6) channel containing patches. Error bars indicate sem

We first established Mg‐ATP concentration IC_50_ curves for wild‐type and five selected SUR2A mutations. K_IR_6.2/SUR2A channels were transiently expressed in HEK293T cells. While using the inside‐out mode of the patch‐clamp electrophysiology method, increasing concentrations of Mg‐ATP were applied to the cytosolic side of the I_KATP_ channels. A dose‐dependent inhibition by Mg‐ATP of I_KATP_ was observed for all channel types for the inward and outward components of the current. Whereas D207E, S1020P, S1054 and R1154Q mutations resulted in a 5‐ to 13‐fold decrease in Mg‐ATP sensitivity (Figure 1, Table 1), the H60Y mutation resulted in no decrease in Mg‐ATP sensitivity compared to WT channels.

**Table 1:** IC_50_ values and Hill slopes for Mg‐ATP dependent inhibition

SUR2A type	Inward current			Outward current			n
	IC_50_ (mmol/L)	Hill slope	*P* value (vs WT)	IC_50_ (mmol/L)	Hill slope	*P* value (vs WT)	
WT	0.14 ± 0.01	−1.21		0.15 ± 0.01	−1.16		8
H60Y	0.14 ± 0.01	−1.05	0.638	0.14 ± 0.01	−1.07	0.697	13
D207E	0.76 ± 0.04	−1.43	**<0.001**	0.71 ± 0.04	−1.26	<**0.001**	7
S1020P	1.93 ± 0.11	−1.84	**<0.0001**	1.83 ± 0.10	−1.57	**<0.0001**	11
S1054Y	1.03 ± 0.07	−1.43	**<0.001**	0.95 ± 0.06	−1.47	**<0.001**	8
R1154Q	0.64 ± 0.11	−1.07	0.051	0.75 ± 0.13	−1.16	**0.038**	6

IC_50_ values are depicted as mean ± sem. P‐values in bold indicate significance.

**Figure 2:**
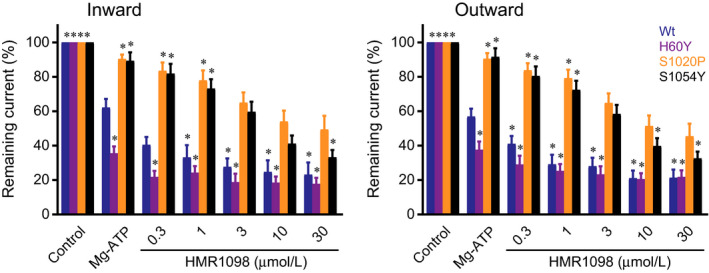
HMR1098 dose‐dependent inhibits K_IR_6.2/SUR2A currents from WT and mutant SUR2A in the presence of 0.15 mmol/L Mg‐ATP. Dose‐dependent (0‐30 μmol/L) effect of HMR1098 application to I_KIR6.2/SUR2A_ (inward at −80 mV inward; outward at + 50 mV) from inside‐out patches containing WT (n = 6), H60Y (n = 4), S1020P (n = 8) or S1054 (n = 9) SUR2A in the continuous presence of 0.15 mmol/L Mg‐ATP. Mean ± sem is depicted, *P < 0.05 vs WT Mg‐ATP

We next determined whether the I_KATP_ inhibitor HMR1098 is able to normalize currents of gain‐of‐function mutants to levels observed in wild‐type channel in the presence of Mg‐ATP only. In the inside‐out mode, we choose to apply increasing concentrations of HMR1098 in the continuous presence of 0.15 mmol/L Mg‐ATP, which is the approximate IC_50_ value of wild‐type channels. Wild‐type and H60Y channels showed a further decrease in currents to approximately 21% of baseline (without Mg‐ATP) at 10 μmol/L HMR1098 (Figure 2). HMR1098 displayed a reduced capacity to inhibit S1020P and S1054Y channels when compared to wild‐type and H60Y channels. Nevertheless, 3 μmol/L HMR1098 brought S1020P and S1054Y channel currents to levels similar as wild‐type channels with 0.15 mmol/L Mg‐ATP alone.

**Figure 3:**
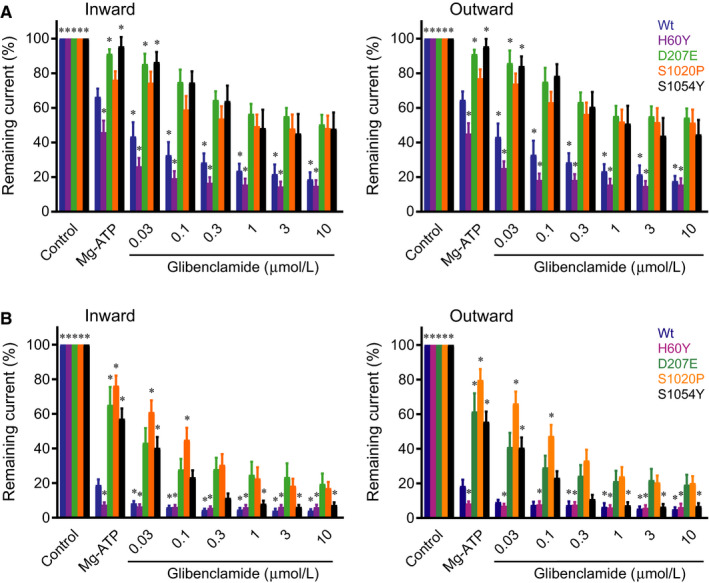
Glibenclamide dose‐dependent inhibits K_IR_6.2/SUR2A currents from WT and mutant SUR2A in the presence of 0.15 mmol/L and 0.5 mmol/L Mg‐ATP. (A, B) Dose‐dependent (0‐30 μmol/L) effect of glibenclamide application to I_KIR6.2/SUR2A_ (inward at −80 mV inward; outward at + 50 mV) from inside‐out patches containing WT (n = 9 and n = 6 for 0.15 and 0.5 mmol/L Mg‐ATP, respectively), H60Y (n = 11, 5), D207E (n = 6, 9), S1020P (n = 10, 9) or S1054Y (n = 7, 7) SUR2A in the continuous presence of 0.15 mmol/L Mg‐ATP (A) or 0.5 mmol/L Mg‐ATP (B). Mean ± sem is depicted, *P < 0.05 vs WT Mg‐ATP

Using a similar approach as for HMR1098, we found that the I_KATP_ inhibitor glibenclamide decreased currents of wild‐type and H60Y channels to approximately 15%‐25% of baseline (without Mg‐ATP) at 1 μmol/L glibenclamide (Figure 3A). Under similar conditions, D207E, S1020P and S1054Y displayed a reduced glibenclamide sensitivity. However, at 1 μmol/L and in the presence of 0.15 mmol/L Mg‐ATP, glibenclamide inhibited these channels to approximately 51%‐56% of baseline values. A further increase in concentration up to 10 μmol/L or 100 μmol/L (D207E) did not cause additional blockage.

To determine whether inhibition capacity by glibenclamide was dependent on Mg‐ATP concentration, similar measurements were performed in the presence of 0.5 mmol/L Mg‐ATP, approximately three times the IC_50_ value for wild‐type channels. In general, all but one, that is S1054Y, channels displayed qualitatively similar responses as seen with 0.15 mmol/L Mg‐ATP, although absolute currents were lower (Figure 3B). In contrast, the S1054Y channel displayed an increased glibenclamide sensitivity. At 1 μmol/L glibenclamide remaining current levels were 7%, which was similar as those of wild‐type and H60Y channels.

### 3.2 Structural interpretation of CS‐causing mutations and drug dependent blockade

**Figure 4:**
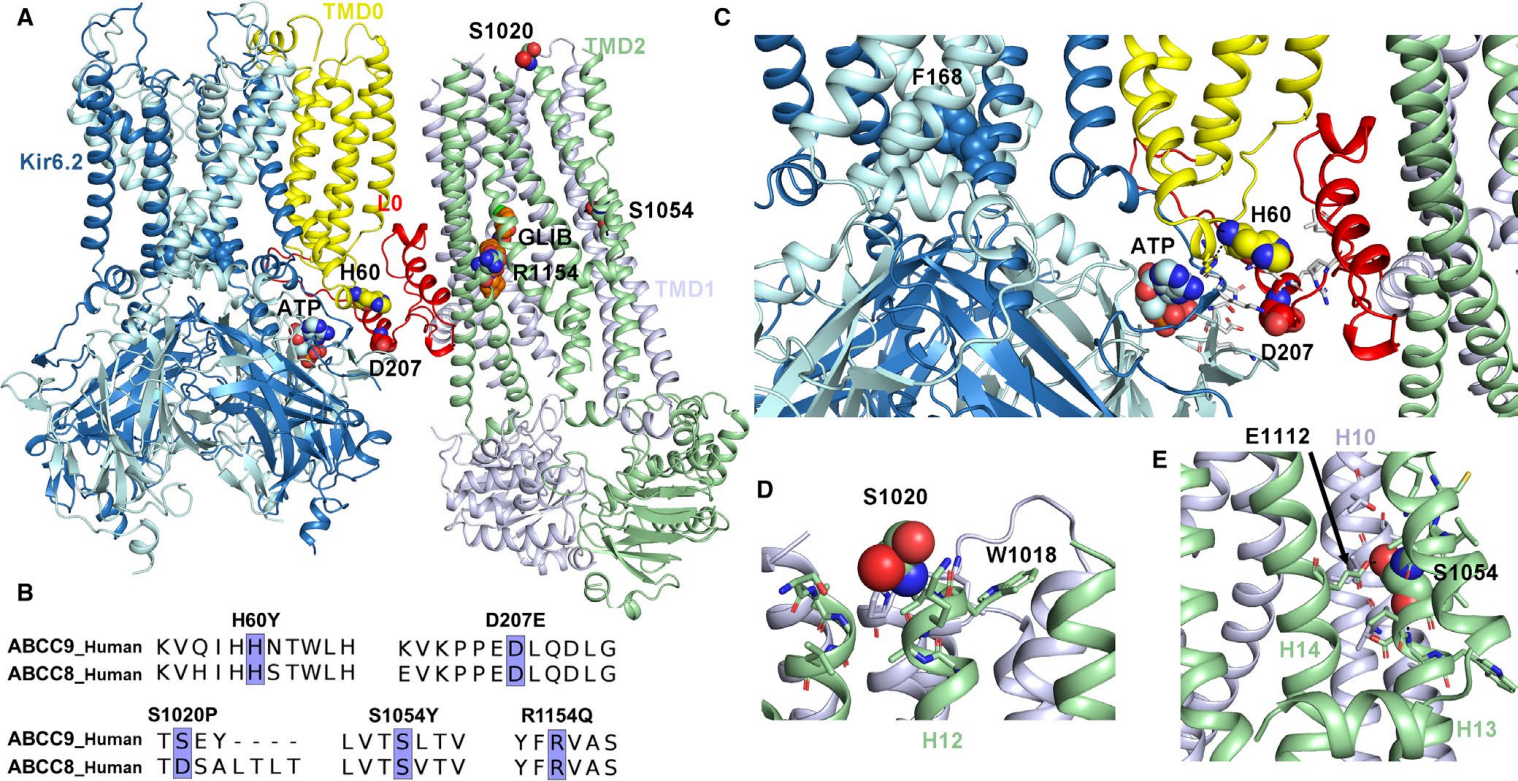
Location of CS mutants in the K_ATP_ channel structure. (A) Side view of a closed K_IR_6.2 channel (coloured blue) with one (out of four) sulphonylurea subunits shown, coloured from yellow, red, light blue and light green (pdb accession no. 6BAA, 3.63 Å resolution). (B) Sequence alignments, indicating conservation between SUR1 and SUR2, with CS mutations shaded in purple. (C) Residues within 4 Å of the H60Y and D207E mutations (spheres), shown as sticks. The ATP binding site is within 10 Å of both mutations; no direct interactions are predicted in the glibenclamide bound, ATP inhibited conformation of K_ATP_ channel. For clarity, also the location of the helix bundle crossing (HBC) gate, with the F168 residues, forming the narrowest part of the closed gate, which is > 37 Å away from H60 and D207, is shown. (D) Residues, surrounding the S1020 residue, located at the extracellular side of helix 12 in TMD2 are shown. (E) S1054, shown in space fill, located in helix 13 of TMD2, is tightly surrounded by residues of helix 10 (TMD1) and helix 14 (TMD2). A hydrogen bond to E1112 is predicted.

The location of the in this study examined CS‐causing mutations in the I_KATP_ channel (pdb: 6BAA) is shown in Figure 4A. H60Y is located in the intracellular region of transmembrane domain 0 (TMD0), a key domain, implicated in gating and trafficking regulation,^26^, ^27^ which connects the SUR protein with the K_IR_6 subunits of the I_KATP_ channel. Mutation D207E is located in the intracellular L0 linker, connecting TMD0 with TMD1. CS mutants S1020P, S1054Y and R1154A are located in the TMD2 region. Except for position S1020, located on the extracellular end of helix 12 (TMD2, Figure 4A,D), all studied CS‐causing mutations are conserved between SUR1 and SUR2 (Figure 4B), which share ~70% sequence identity.

**Figure 5:**
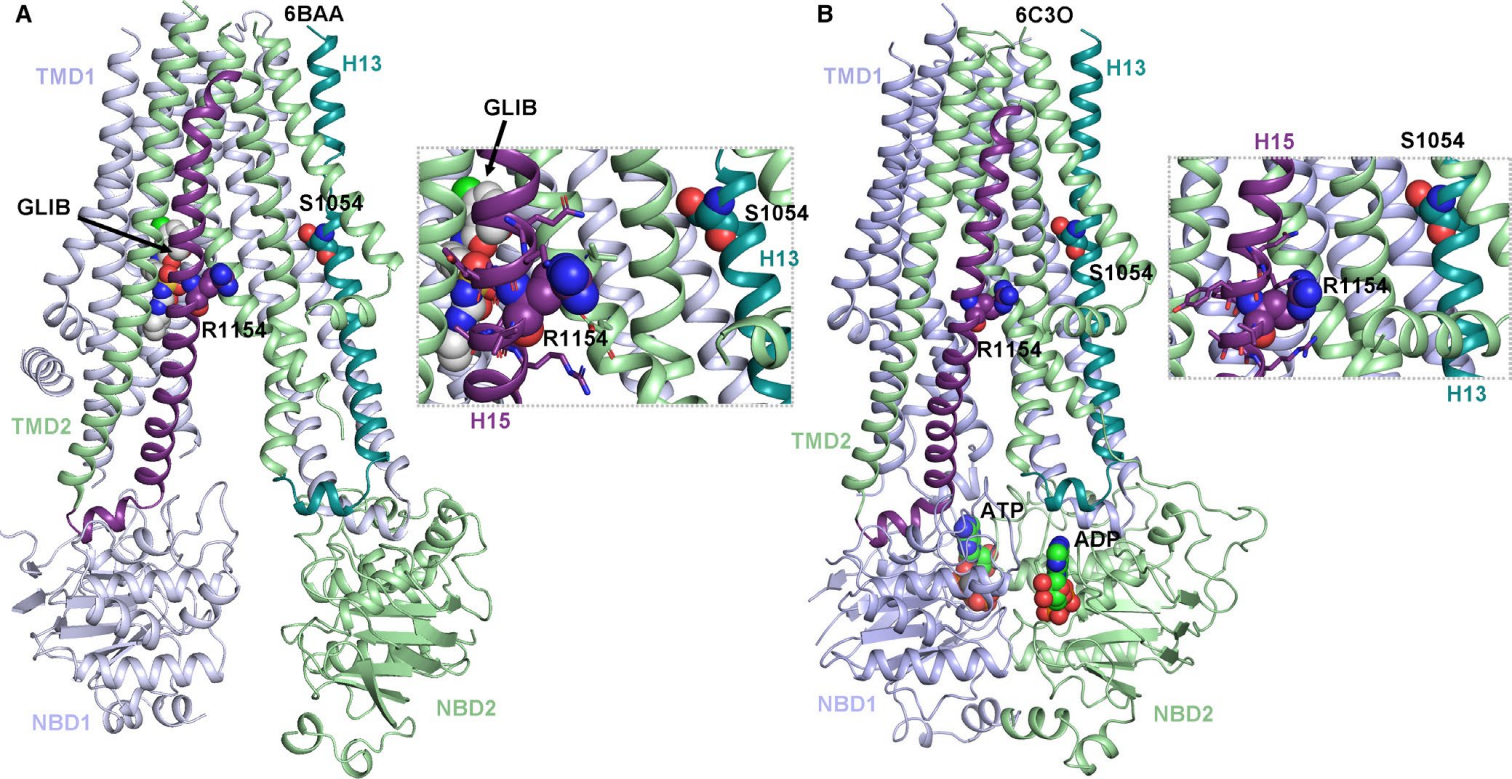
Structural comparison of the ABC core structure of SUR1 in different conformations. (A) Structure of the TMD1‐NBD1‐TMD2‐NBD2 complex in the glibenclamide (GLIB) inhibited state. TMD1 and NBD1 are coloured light blue, while TMD2 and NBD2 are coloured light green. The insets show residues within 4 Å of CS mutations S1054Y (top) and R1154Q (bottom). (B) The same SUR domains as in (A), in the Mg nucleotide activated conformation is shown. ADP and ATP molecules bound to the catalytic and degenerate sites, are shown as spheres. The insets again show residues within 4 Å of CS mutations S1054Y (top) and R1154Q (bottom)

To obtain insights into possible gating‐associated conformational changes of the CS mutants, we compared the two cryo‐EM structures with the highest resolution. One structure was solved in the presence of the inhibitory sulphonylurea drug glibenclamide (pdb: 6BAA, 3.63 Å 23), while the other structure contains ATP bound to the inhibitory site on K_IR_6.2 and activating Mg nucleotides in both nucleotide binding domains of SUR1 (pdb: 6C3O, 3.9 Å 24). Unfortunately, the intracellular loop regions containing CS mutations H60Y and D207E are not resolved in the Mg‐nucleotide activated I_KATP_ channel structure (pdb: 6C3O). Further, due to the lack of sequence conservation (see alignment in Figure 4B) and missing residues in the immediate vicinity of position S1020, functional interpretations are not possible. S1054Y, located in helix 13 might form a hydrogen bond with E1112 of helix 14, in the glibenclamide‐inhibited 6BAA structure (non‐dimerized NBDs, Figure 4A,E). Three residues upstream of the CS mutant, helix 13 contains a stretch of unfolded residues (1048‐1051), while in the nucleotide bound structure this stretch is helical (compare Figure 5A,B). As in classical ABC transporters, conformational changes induced by nucleotide binding and dimerization of the NBD domains are transduced to the transmembrane helices of SUR via coupling helices (IH3 and IH4 are marked in Figure 5A,B). However, dimerization in the currently available cryo‐EM structure did not lead to an outward facing conformation of the transporter, in agreement with the lack of any known transport function for SURs. It is currently unclear if the SUR subunits can adopt this outward‐open conformation although a recent study suggests that this might indeed be possible.^28^

Since helix 13 is directly connected to coupling helix IH3, which transmits conformational changes induced by Mg nucleotide binding, it is perhaps not surprising that mutation S1054Y does influence the stimulatory effect of Mg nucleotides, as seen in our experiments (Figure 1, Table 1). In silico mutating S1054 to tyrosine suggests that the bulky tyrosine side‐chain would not fit into the cryo‐EM structures without inducing steric clashes (not shown). This suggest that the mutation will induce local conformational changes, most likely followed by currently unpredictable global conformational changes.

Residue R1154 is located in helix 15, in the second half of the TMD2 module, which is connected to the degenerate site of the NBD, via coupling helix IH4, as shown in Figure 5B.

It might be speculated that this mutation induces similar disturbances to channel gating as observed for the S1054 mutant, located in a comparable position (Figure 5). Indeed, our functional data reveal that the R1154Q mutation results in decreased Mg‐ATP sensitivity (Figure 1). Interestingly, R1154 is in close vicinity of the glibenclamide binding site (Figure 5A); however, the mutant is located on the opposite site of the binding site, facing away from glibenclamide, thus no direct interaction with the drug is possible.

## 4 DISCUSSION

Cantú has been attributed to *ABCC9* gain‐of‐function mutations, more specific (Mg)‐ATP mediated inhibition is affected resulting in higher I_KATP_ densities at identical Mg‐ATP concentrations.^4^ But the underlying mechanisms are mutation specifically and include enhanced Mg‐ATP/ADP activation and increased intrinsic open probability.^7^, ^29^ Indeed, D207E, S1020P, S1054Y and R1154Q displayed gain‐of‐function characteristics in response to Mg‐ATP. IC_50_ values of R1154Q (0.64 ± 0.11 mmol/L and 0.75 ± 0.13 mmol/L for inward and outward current respectively) were in the same order as previously reported for the same SUR2A mutation (0.88 ± 0.19, 0.76 ± 0.12).^4^ In our assays, the H60Y mutation does not display gain‐of‐function behaviour in response to Mg‐ATP application compared with wild‐type channels. The clinical characteristics of the H60Y patients however are indistinguishable from many other CS patients.^2^, ^4^ Besides altered current characteristics, gain‐of‐function may also be achieved for example by increased channel expression levels, enhanced forward trafficking of decreased channel degradation, that might be difficult to fully replicate in an ectopic expression system. Furthermore, we cannot exclude the possibility that in combination with K_IR_6.1, instead of K_IR_6.2, the H60Y mutation will induce gain‐of‐function behaviour with respect to Mg‐ATP sensitivity. Also, alterations in pH‐dependent effects on I_KATP_ could result from the H60Y mutation. Therefore, the mechanism by which H60Y results in CS requires further investigation and may require other channel subunits, cell systems and in vivo models.

In the absence of nucleotides, D207E sensitivity for glibenclamide was in the nanomolar range (42.8 ± 11.4 nmol/L) and similar as for wild‐type channels (29.5 ± 11.1 nmol/L).^29^ However, in the presence of nucleotides (0.15 mmol/L Mg‐ATP and 0.5 mmol/L Mg‐ATP) and thus better mimicking the physiological conditions, glibenclamide sensitivity is blunted and now is in the micromolar range (Figure 3). Previously, we found a similar mutation‐dependent decrease in glibenclamide sensitivity for the CS V65M mutation in K_IR_6.1.^30^ There is an elegant, but not fully understood, interplay between glibenclamide‐mediated block, Mg‐ATP/ADP mediated activation at the SUR2 subunit and (Mg)‐ATP mediated block at the K_IR_6.2 subunit.^21^ Interestingly, raising Mg‐ATP from 0.15 mmol/L to 0.5 mmol/L specifically potentiates the blocking capacity of glibenclamide for the S1054Y channel. At 0.15 mmol/L glibenclamide responses are similar as seen for D207E and S1020P, whereas at 0.5 mmol/L Mg‐ATP S1054Y goes with WT and H60Y from glibenclamide concentrations of 0.3 μmol/L and higher. We propose that this mutation may be helpful in determining the mechanistic interplay of these inhibiting and activating actions of the underlying compounds.

The clinical significance of glibenclamide and HMR1098 for Cantú patient treatment obviously depends on the sensitivity of the mutant channels for these blockers and drug‐safety relationships. Glibenclamide has a therapeutic range of approximately 100‐400 nmol/L 31, 32 and becomes toxic from 1.2 μmol/L.^32^, ^33^ Therefore, even in the presence of Mg^2+^ and nucleotides that to some extent mimic the intracellular conditions, glibenclamide concentrations required to achieve a completely normalized K_IR_6.2/SUR2A current with respect to wild‐type conditions are most likely beyond the therapeutic and safe range. The clinical benefits of partial normalization of K_IR_6.2/SUR2A current remains to be determined. HMR1098, which is the sodium salt of HMR‐1883 that is also known as Clamikalant, has been developed as a cardioselective blocker of I_KATP_ channels for the purpose of antiarrhythmic treatment. Clamikalant was discontinued after phase II trials.^34^, ^35^ Therefore, there is still requirement for new pharmacotherapeutics for CS patients. Whereas this proof‐of‐concept work indicates feasibility of pharmacological correction of CS‐associated gain‐of‐function of I_KATP_ channels, any conclusions on clinical efficacy have to await further studies.

K_ATP_ channel cryo‐EM structures provided us with some insights in drug‐channel interactions and the effects several CS‐associated mutations have on such interactions as shown here. It should be kept in mind, however, that the structure of SUR2A will be different from SUR1 in certain aspects, as indicated by functional differences (eg 36, 37). Furthermore, the lack of sufficient resolution, in particular of the SUR subunits, hampers a more detailed interpretation of the human disease mutants. Thus, further structures of I_KATP_ channels, preferably containing SUR2A subunits and more gating intermediates, combined with molecular dynamics simulations will be needed to understand the structural basis of gating changes induced by CS mutants in more detail. These combinations of structural insight and patch‐clamp electrophysiology will assist future rationalized drug design to address CS.

In this study, for technical reasons, measurements were limited to combinations of K_IR_6.2 and SUR2A (WT and CS mutations), whereas in vivo I_KATP_ channels may also exists as heteromeric K_IR_6.1/K_IR_6.2 tetramers and CS SUR2A protein mutations mostly occur in K_IR_6.1/SUR2 channels.

## DATA SHARING

Data available on request from the authors.

## CONFLICT OF INTEREST

The authors declare no conflict of interest.

## AUTHOR CONTRIBUTIONS

MJCH and KD performed research; MJCH, KD, MQ, ASW and XC analysed the results. MvdH, GvH and ASW designed the study. MvdH and ASW wrote the paper. All the authors reviewed the manuscript.
